# Treatment-Emergent Co-Morbidities and Survival in Patients With Metastatic Castration-Resistant Prostate Cancer Receiving Abiraterone or Enzalutamide

**DOI:** 10.3389/fphar.2021.669236

**Published:** 2021-05-18

**Authors:** Yi-Ting Lin, Yen-Chun Huang, Chih-Kuan Liu, Tian-Shyug Lee, Mingchih Chen, Yu-Ning Chien

**Affiliations:** ^1^ Graduate Institute of Business Administration, College of Management, Fu Jen Catholic University, New Taipei City, Taiwan; ^2^ AI Development Centers, Fu Jen Catholic University, New Taipei City, Taiwan; ^3^ Department of Urology, St. Joseph Hospital, Yunlin County, Taiwan; ^4^ Department of Urology, Fu Jen Catholic University Hospital, New Taipei City, Taiwan; ^5^ College of Medicine Fu Jen Catholic University Master Program of Big Data Analysis in Biomedicine, New Taipei City, Taiwan

**Keywords:** national health insurance research database, metastatic castration resistant prostate cancer, charlson comorbidity index, abiraterone, enzalutamide, enzalutamide overall survival

## Abstract

Secondary hormone therapy, abiraterone and enzalutamide, has improved outcomes for metastatic castration-resistant prostate cancer (mCRPC) and prolonged patients’ lives significantly. Various studies have compared the cancer-related outcomes, adverse effects, and drug-induced comorbidities in patients with mCRPC who are treated with abiraterone or enzalutamide. However, few studies have explored associations between survival and comorbidities or comprehensive analyzed newly developed comorbidities during and after secondary hormone therapy. We attempted to clarify whether the Charlson comorbidity index (CCI) overall or itemized is predictive for overall survival, and we compared newly developed comorbidities between abiraterone and enzalutamide groups. We extracted data about expenses and comorbidities for patients who have mCRPC, received abiraterone and enzalutamide and met pre-examination operation criteria between September 2016 and December 2017 from the Taiwan National Health Insurance database. A total of 1153 patients with mCRPC who received abiraterone (*n* = 782) or enzalutamide (*n* = 371) with or without previous chemotherapy were included. We used the propensity score to match confounding factors, including age, pre-existing comorbidities, and precipitating factors for comorbidity (e.g., hypertension, hyperlipidemia), to eliminate selection bias in the comparison of newly developed comorbidities. Cox regression analysis was used for overall survival. We found that enzalutamide is superior to abiraterone with regard to overall survival. Our study revealed that there is no statistically significant difference in development of new comorbidities between abiraterone and enzalutamide group. Moreover, the CCI score, rather than any single item of the CCI, was a statistically significant predictor for overall survival.

## Introduction

The new generation of secondary hormone therapy has improved the outlook for patients with metastatic castration-resistant prostate cancer (mCRPC). This therapy has improved cancer-related outcomes and prolonged patients’ lives significantly (Fizazi et al., 2012; Scher et al., 2012). The two medications approved in this class are abiraterone and enzalutamide. The clinical indications of these two medications are quite similar. However, there is no clear guideline that provides an accepted recommendation for selecting between them. A comparison of the outcomes and adverse effects related to these two medications will help select the best medication, and this comparison is being extensively discussed. Several studies have compared cancer-related outcomes, including disease-specific survival, progression-free survival, and initial response rate; other studies have focused on comparing adverse effects between the two medications (Kang et al., 2017; Chung et al., 2019; Komura et al., 2019; Markowski et al., 2021). A systematic review and a network meta-analysis of the treatments for chemotherapy-naive patients revealed that enzalutamide was superior to abiraterone/prednisone and sipuleucel-T with regard to radiographic progression-free survival (McCool et al., 2018). Another meta-analysis that focused on the effectiveness and safety outcomes of abiraterone vs. enzalutamide in patients with mCRPC demonstrated that enzalutamide was associated with a higher prostate-specific antigen (PSA) response rate than abiraterone in patients with mCRPC, and no significant difference was found between the two groups with regard to overall adverse effects (Wang et al., 2020).

However, to our knowledge, no head-to-head randomized, controlled trial has compared these two medications, except Khalaf and colleagues’ work which aimed at comparison of sequences of medications. Hara et al. (2018) designed a study protocol for a multicenter, randomized, phase 3 trial to compare these two medications. The results of this trial are still pending. The outcomes of the use of these two medications extracted from big medical database still have roles to generate evidences.

Many studies have compared adverse effects between these medications. Lee et al. conducted a meta-analysis of data from 7103 patients across seven randomized, controlled trials. The analysis showed that abiraterone had a higher probability of cardiac disorders than enzalutamide, whereas enzalutamide had a higher probability of hypertension than abiraterone (Lee et al., 2020). The AQUARIUS study was a prospective, 12-months, observational study in patients with mCRPC from Denmark, France, and the United Kingdom. The study suggested an advantage of abiraterone acetate plus prednisone over enzalutamide with regard to fatigue and cognitive function (Thiery-Vuillemin et al., 2020). A meta-analysis by Khalaf et al. (2019) demonstrated differential adverse effects profile in patients with mCRPC who were treated with abiraterone or enzalutamide. They pointed out that abiraterone was associated with an increased risk of cardiovascular events, whereas enzalutamide was associated with an increased risk of fatigue (Moreira et al., 2017). Thus, the risks of cognitive impairment, fatigue and cardiovascular events differed significantly between these two medications.

Comorbidities have become important predictive factors for survival in patients with prostate cancer. The Charlson comorbidity index (CCI) has been an important prognostic factor for long-term survival outcomes in Korean men with prostate cancer after radical prostatectomy (Lee et al., 2014). Knipper et al. used the CCI to build a nomogram for the prediction of 10-years life expectancy in candidates for radical prostatectomy (Knipper et al., 2019). An association between comorbidities and survival may exist in patients with mCRPC. Goyal et al. attempted to use the CCI and hypertension as indicators of survival in men with mCRPC. They found that hypertension alone and hypertension combined with CCI were borderline significantly associated with overall survival on both univariable and multivariable analyses (Goyal et al., 2014). However, few if any studies were designed to survey the effects of comorbidity on overall survival and disease-specific survival in patients with mCRPC who were treated with abiraterone or enzalutamide. Furthermore, to our knowledge, no literature has comprehensively analyzed the effect of the most of common comorbidities on survival in a single dataset. If comorbidity is a significant predictor for overall and disease-specific survivals, then the comparison of survival is biased when comorbidity differences between study arms are ignored. Therefore, we should perform propensity score matching to ensure that baseline comorbidity measures are equal during an observational study.

Researchers believe that some comorbidities can be induced by androgen deprivation therapy. One study revealed an increased risk of ischemic stroke after androgen deprivation therapy for prostate cancer in a Chinese population living in Hong Kong (Teoh et al., 2015). Another study demonstrated an increased risk of diabetes among patients who received primary androgen deprivation therapy for clinically localized prostate cancer (Tsai et al., 2015). Yet another study confirmed the association between androgen-deprivation therapy and metabolic syndrome in men with prostate cancer (Harrington et al., 2014). An Australian, population-based, cohort study showed that hazard ratios for cardiovascular conditions and depression were highest in the first year after androgen deprivation therapy and declined over time (Ng et al., 2018). Lu et al. (2019) discovered an associations between peripheral thromboembolic vascular disease and androgen deprivation therapy in Asian patients with prostate cancer. Researchers also believe that some comorbidities can be induced by abiraterone. Tucci et al. (2019) reported that abiraterone plus prednisone therapy may cause severe hypoglycemia when administered to patients with prostate cancer and type 2 diabetes who are receiving glucose-lowering agents. Another report suggested monitoring of blood pressure and cardiovascular events during abiraterone treatment according to its main finding of incremental risk of hypertension (Zhu and Wu, 2019). Colomba et al. (2020) reported an rare increase in liver enzymes during treatment with abiraterone acetate in their study population with metastatic prostate cancer. Singh et al. (2018) also reported the development of abiraterone-associated fulminant liver failure in one patient.

Pilon et al. (2017) conducted a large observational study and found that, among patients with metastatic prostate cancer, abiraterone acetate, compared with enzalutamide or chemotherapy, was associated with a significantly lower likelihood of having a central nervous system event. Specific adverse effects tend to develop in patients with specific predisposing conditions; for example, prednisolone induces high blood sugar in patients with pre-existing diabetes mellitus type 2 (Bonaventura and Montecucco, 2018); medication can induce cardiovascular events in patients with pre-existing hypertension or hyperlipidemia (Francula-Zaninovic and Nola, 2018). Therefore, when selecting medications, it is reasonable to prescribe medications in patients with pre-existing conditions that would not increase the risk of specific adverse effects, rather than completely concentrating on survival to guide treatment choice. To guide treatment determinations, studies designed to compare outcomes among therapeutic protocols and adverse effects are needed.

The CCI is a widely used and well-accepted tool for comorbidity assessment (Charlson et al., 1994). Charlson comorbidity is closely associated with life expectancy and could serve as a significant predictor of survival in patients with prostate cancer. Items in the CCI include the most common comorbidities and could be used in clinical practice as a checklist for collecting comorbidity history. The Taiwan National Health Insurance Research database (NHIRD) has high-quality comorbidity data, and studies in orthopedic, psychological, endocrinological, and intensive care territories have successfully extracted Charlson comorbidity data to determine any association between the CCI and the studied conditions (Knipper et al., 2019; Hsu et al., 2020; Tang et al., 2020; Tseng et al., 2020).

In qualitative research, drug selection almost always involves maximal avoidance of adverse effects and minimization of treatment failure (Maxwell, 2016). Therefore, to fulfill these two principles in the management of mCRPC with new-generation secondary hormone therapy, we compared treatment outcomes and treatment-emergent morbidities of this therapy. We had two goals in this study: The first goal was to compare cancer-related outcomes between groups treated with abiraterone and with enzalutamide after adjusting the inequality of the comorbidity status between groups, using data extracted from the NHIRD. We used a propensity score to match factors that significantly influenced overall survivals (e.g., comorbidities, follow-up times, and durations of medication use) between the groups. We used Cox regression analysis to identify the risk factors for mortality, as reflected by overall survivals. The second goal was to compare newly developed comorbidities during treatment with abiraterone or with enzalutamide. We used CCI items as the standard to collect comorbidity data about every patient. The details of data collection and statistical methods are described in *Materials and Methods*.

## Materials and Methods

### Data Source

The NHIRD contains individual clinical and in-hospital data information (i.e., disease profiles, medical costs, and diagnostic codes) for more than 99% of the population. The diagnostic codes, based on the 10th revision of the International Classification of Diseases (ICD-10-CM), were fully adopted as of January 1, 2016. Files from the Taiwan Cancer Registry (TCR) contain detailed laboratory values and detailed clinical information about patients. The TCR central office provides complete details of procedures, which ensures the accuracy of cancer registration data. If data have errors, they are sent back to hospitals for checks and corrections. According to Charlson et al. (1994), the TCR in 2015 was one of the highest-quality cancer registry files in Asia and the world; it currently provides 90% coverage of cancer cases in Taiwan. The NHIRD provides anonymous demographic and administrative information; thus, the requirement to obtain informed consent for this study was waived. This study was fully approved by the Institutional Review Board of Fu-Jen University in Taiwan (No. C108121).

### Defining the Target Population

The target population in this study was defined as patients with mCRPC who started abiraterone or enzalutamide treatment between September 2016 and the end of 2017, with or without previous chemotherapy. Taiwan national health insurance began to cover abiraterone payments in December 2014 and enzalutamide payments in September 2016. A pre-review counter is required for every payment of these two medications. These two medications shared similar strict criteria for pre-review counter, including the following (Fizazi et al., 2012): Eastern Cooperative Oncology Group score of ≤2 (Scher et al., 2012); poor response to at least two courses of docetaxel; and (Chung et al., 2019) combination of abiraterone with prednisone or prednisolone. Every patient had to satisfy the first two criteria before they could obtain payment from national health insurance, and patients treated with abiraterone also had to satisfy the third criterion. The pre-review counter for every payment is peer-reviewed by experienced urologists in Taiwan. These processes might have increased the rigor of patient enrollment in this study. Taiwan national health insurance has covered the payment of these two medications in chemotherapy-naive mCRPC since September 2017. The two study medications shared the same criteria for pre-review counter in chemotherapy-naive patients. Therefore, we screened the outpatient expense file and inpatient expense file with the medication charge codes (BC26139100 and BC27291100 for abiraterone, and BC26634100 for enzalutamide) to identify the patients who received abiraterone or enzalutamide before December 2017; then, we excluded patients who received abiraterone and enzalutamide before September 2016. The remaining patients composed our target population.

### Data Collection and Variable Defining

**FIGURE 1:**
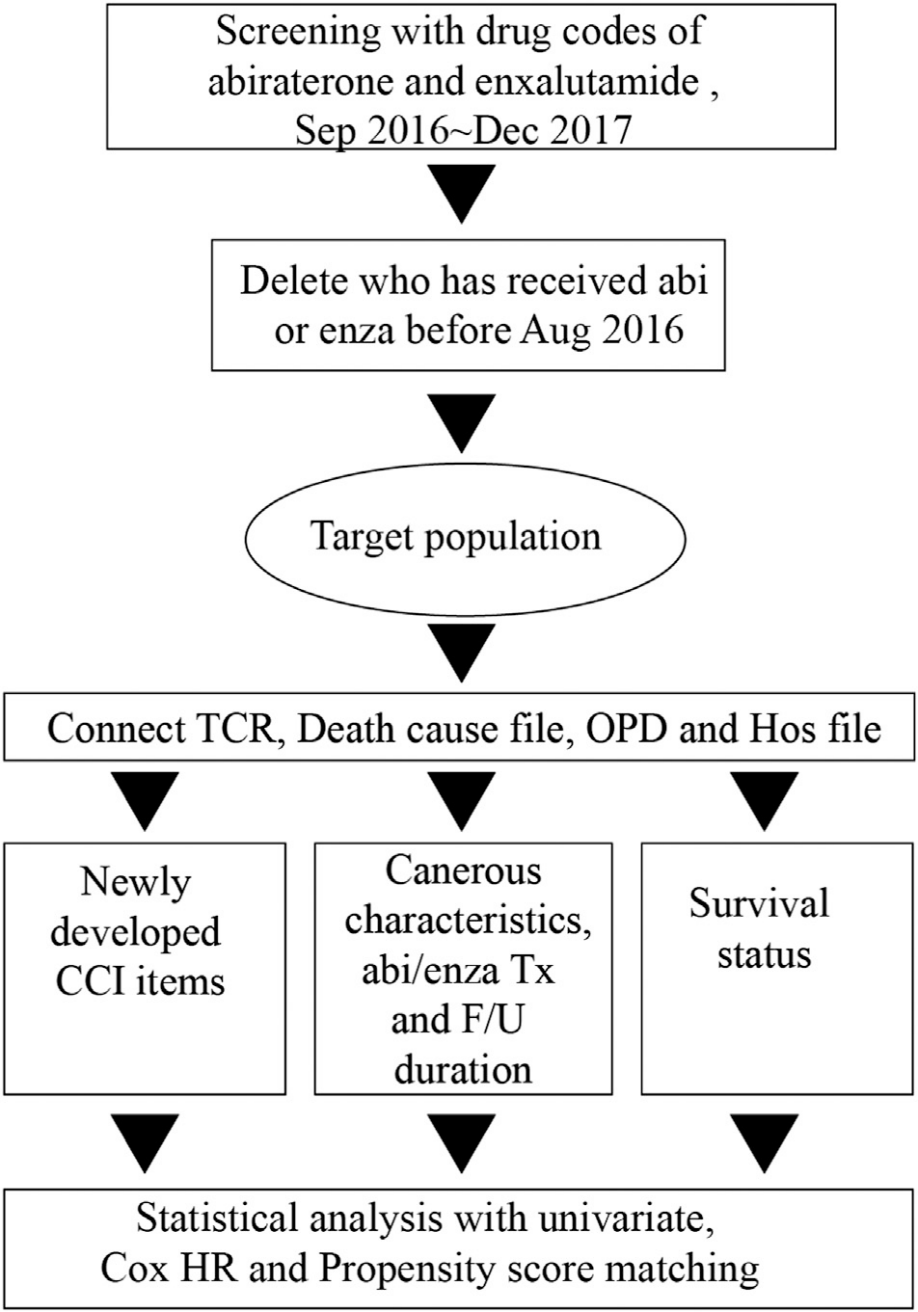
Flowchart of subject enrollment and data collection.

After the study population was determined, we connected data from the TCR file, the cause-of-death file, and the inpatient file for each patient. We obtained cancer-related characteristics, cause of death, comorbidity status, and personal history (e.g., smoking history or alcohol consumption). The data collection period covered September 2016 to December 2017. We also obtained duration of follow-up and duration of medication use. We observed any newly developed comorbidities for every patient in the study population. First, we determined the presence or absence of comorbidities by screening ICD-9 and ICD-10 codes for items included in the CCI, such as myocardial infarction, congestive heart failure, peripheral vascular disease, cerebrovascular disease, dementia, chronic pulmonary disease, rheumatic disease, mild liver disease, diabetes without chronic complication, diabetes with chronic complication, hemiplegia or paraplegia, renal disease, any malignancy (including lymphoma and leukemia) except malignant neoplasm of skin, moderate or severe liver disease, metastatic solid tumor, and HIV/AIDS, from inpatient and outpatient files of the NHIRD for every patient (Zhu and Wu, 2019). The newly developed comorbidities were defined, in any individual patient, as those extracted before the end of 2018 minus those extracted before the respective start date (or a surrogate) of abiraterone or enzalutamide treatment (Figure 1).

### Statistical Analysis

The demographic characteristics of the study populations were compared with the chi-square test to calculate frequencies or percentages of categorical variables, and the *t*-test was performed to determine the mean difference for continuous variables. To consider the potential confounding factors in this research, both cohorts were matched 1:1 in a propensity score method based on a logistic regression model; variables included baseline age, CCI items, chemotherapy, grade, duration of follow-up, duration of medication use, and precipitating factors of cerebrovascular disease and cardiovascular disease (e.g., hypertension, hyperlipidemia, diabetes mellitus) to generate the after-matching groups. Demographic and clinical characteristics are listed in Table 1. We used the after-matching groups to compare newly developed comorbidities (Table 2). The risk of overall mortality was an important consideration in this study; we used a multivariable Cox proportional hazard models for the sensitivity analysis. The results were expressed as adjusted hazard ratios (aHRs) and 95% confidence intervals (CIs). Kaplan-Meier curves and log-rank tests were performed to obtain the curves of cumulative mortality incidences for the enzalutamide and abiraterone groups. All statistical analyses were performed with SAS 9.4 (SAS Institute, Cary, NC) and R software (version 3.4.3; Project for Statistical Computing, Vienna, Austria). Statistical significance was two tailed and set at *p* < 0.05.

**TABLE 1:** Demographic and clinical characteristics of patients.

	Before matching					After matching				
Variables	Abiraterone (*n* = 782)		Enzalutamide (*n* = 371)		*p*	Abiraterone (*n* = 365)		Enzalutamide (*n* = 365)		*p*
	**N**	**%**	**N**	**%**		**N**	**%**	**N**	**%**	
**Chemotherapy**										
Received	474	60.61	168	45.28	**<0.0001**	165	45.21	168	46.03	0.824
naïve	308	39.39	203	54.72		200	54.79	197	53.97	
**Age group**										
<65	264	33.76	102	27.49	**0.033**	115	31.51	101	27.67	0.256
≥65	518	66.24	269	72.51		250	68.49	264	72.33	
Age (mean SD)	68.70 (9.05)		70.41 (9.01)		**0.003**	69.58 (9.12)		70.41 (9.03)		0.219
**Grade**										
1	15	1.92	13	3.5	0.098	10	2.74	13	3.56	0.491
2	108	13.81	61	16.44		55	15.07	61	16.71	
3	571	73.02	267	71.97		259	70.96	261	71.51	
Unknow	88	11.25	30	8.09		41	11.23	30	8.22	
**Precipitating conditions for comorbidities**										
Hypertension	413	52.81	219	59.03	**0.048**	211	57.81	216	59.18	0.707
Diabetes mellitus	218	27.88	113	30.46	0.366	110	30.14	113	30.96	0.809
Hyperlipidemia	440	56.27	226	60.92	0.135	200	54.79	222	60.82	0.099
CHF	67	8.57	39	10.51	0.286	33	9.04	39	10.68	0.456
CKD	155	19.82	77	20.75	0.712	78	21.37	73	20	0.648
Stroke	189	24.17	88	23.72	0.868	86	23.56	85	23.29	0.930
PVD	71	9.08	31	8.36	0.686	37	10.14	30	8.22	0.369
ACS	63	8.06	35	9.43	0.433	28	7.67	34	9.32	0.426
COPD	342	43.73	159	42.86	0.779	171	46.85	156	42.74	0.264
Average F/U (months)	14.53 (7.97)		12.84 (5.52)		**<0.0001**	12.41 (6.82)		12.93 (5.48)		0.261
Median of F/U (months)	13.97		13.43			13.03		13.47		
Average duration of medication use (months)	4.71 (4.52)		2.74 (2.86)		**<0.0001**	2.80 (2.90)		2.78 (2.86)		0.929
Median duration of medication use (months)	2.8		1.87			1.87		1.87		
Overall mortality	407	52.05	151	40.7	**0.0003**	196	53.7	148	40.55	**0.0004**
**CCIS scores**										
0	31	3.96	9	2.43	**0.001**	19	5.21	9	2.47	**0.046**
1	0	0	0	0		0	0	0	0	
2	26	3.32	15	4.04		11	3.01	15	4.11	
3	30	3.84	31	8.36		17	4.66	30	8.22	
4	28	3.58	26	7.01		15	4.11	26	7.12	
5	30	3.84	16	4.31		17	4.66	16	4.38	
6+	637	81.46	274	73.85		286	78.36	269	73.7	
Mean (SD)	9.15 (3.89)		8.62 (4.10)		**0.034**	8.86 (4.05)		8.61 (4.11)		0.410
**CCI items**										
CPD	346	44.25	157	42.32	0.538	171	46.85	154	42.19	0.206
Cerebrovascular disease	189	24.17	88	23.72	0.868	86	23.56	85	23.29	0.930
Moderate or severe renal disease	247	31.59	108	29.11	0.395	113	30.96	104	28.49	0.466
PVD	71	9.08	31	8.36	0.686	37	10.14	30	8.22	0.370
Ulcer disease	375	47.95	182	49.06	0.726	175	47.95	179	49.04	0.767
CHF	67	8.57	39	10.51	0.286	33	9.04	39	10.68	0.456
Diabetes	218	27.88	113	30.46	0.366	110	30.14	113	30.96	0.810
Mild liver disease	120	15.35	58	15.63	0.899	60	16.44	58	15.89	0.841
MI	38	4.86	26	7.01	0.137	20	5.48	25	6.85	0.442
CTD	7	0.9	3	0.81	1.000	5	1.37	3	0.82	0.725
Diabetes with end organ damage	96	12.28	45	12.13	0.943	43	11.78	45	12.33	0.820
Dementia	29	3.71	16	4.31	0.621	15	4.11	16	4.38	0.854
Hemiplegia	7	0.9	8	2.16	0.095	5	1.37	8	2.19	0.401
Moderate severe liver disease	3	0.38	4	1.08	0.156	0–5%		0–5%		0.373
AIDS	17	2.17	7	1.89	0.750	6	1.64	7	1.92	0.780

CPD, Chronic pulmonary disease; PVD, Peripheral vascular disease; CTD, Connective tissue disease; MI, Myocardial infarct; CHF, Congestive heart failure; CKD, Chronic Kidney disease; ACS, Acute Coronary Syndrome. *Grade 1 represents Gnleaso score 4 and 5; 2 represents 6,7; 3 represents 8,9,10. Bold values mean significantly related.

**TABLE 2:** Newly developed comorbidities.

	Before matching					After matching				
Variables	Abiraterone (*n* = 782)		Enzalutamide (*n* = 371)		*p*	Abiraterone (*n* = 365)		Enzalutamide (*n* = 365)		*p*
	**N**	**%**	**N**	**%**		**N**	**%**	**N**	**%**	
Cerebrovascular disease	22	2.81	12	3.23	0.693	9	2.47	12	3.29	0.507
PVD	6	0.77	0–5%		1.000	3	0.82	0–5%		1.000
Ulcer disease	23	2.94	14	3.77	0.454	13	3.56	14	3.84	0.845
CHF	24	3.07	12	3.23	0.880	9	2.47	11	3.01	0.650
CKD	25	3.20	7	1.89	0.206	10	2.74	7	1.92	0.462
MI	5	0.64	0–5%		1.000	4	1.1	0–5%		0.686
Diabetes	30	3.84	8	2.16	0.136	15	4.11	8	2.19	0.138
Dementia	12	1.53	5	1.35	0.806	9	2.47	5	1.37	0.280
ACS	6	0.77	3	0.81	1.000	4	1.1	0–5%		1.000

CPD, Chronic pulmonary disease; PVD, Peripheral vascular disease; CTD, Connective tissue disease; MI, Myocardial infarct; CHF, Congestive heart failure; CKD, Chronic Kidney disease; ACS, Acute Coronary Syndrome. Bold values mean significantly related.

## Results

### Demographic Characteristics of Study Population

Overall, 1153 individuals were divided into two different groups: abiraterone (*n* = 782) and enzalutamide (*n* = 371). The age, status of chemotherapy, hypertension status, duration of follow-up, and duration of medication use differed between both cohorts (Table 1). Thus, we used the propensity score method to match the groups. After matching, there was no significant difference between the abiraterone (*n* = 365) and enzalutamide (*n* = 365) cohorts with regard to characteristics or comorbidities. The abiraterone group had higher rates of overall mortality (53.7 vs. 40.55%, *p* = 0.0004) and more CCI scores of ≥6 (78.36 vs. 73.70%, *p* = 0.046). These results, after propensity score matching to adjust for age, chemotherapy use, and hypertension, suggested that overall survival was better with enzalutamide than with abiraterone.

### Outcomes of Newly Developed Comorbidities

According to the study design, we attempted to match factors which affects treatment-emergent co-morbidities, including, age, cancer-related characteristics, pre-existing comorbidities, precipitating factors of comorbidities, etc. After univariate analysis, we found age, chemotherapy, hypertension, duration of follow-up, and duration of medication use were statistical-significantly unequal between groups. Therefore, we matched them with propensity score. No statistically significant difference was observed in the newly developed comorbidities after matching; included cerebrovascular disease (abiraterone vs enzalutamide: 2.47 vs. 3.29%, *p* = 0.507), peripheral vascular disease (0.82% vs 0–5%, *p* = 1.000), peptic ulcer disease (3.56 vs. 3.84%, *p* = 0.845), congestive heart failure (2.47 vs. 3.01%, *p* = 0.650), chronic renal disease (2.74 vs. 1.92%, *p* = 0.462), myocardial infarction (4 vs. 05%, *p* = 0.686), diabetes mellitus (4.11 vs. 2.19%, *p* = 0.138), dementia (2.47 vs. 2.19%, *p* = 0.280), and acute coronary syndrome (1.1% vs. 0–5%, *p* = 1.000).

### Risk of Overall Mortality With Different aHR Models

We established two Cox regression models with two different variable sets. Variables for model 1 were abiraterone or enzalutamide use, age, receipt of chemotherapy or not, tumor grade, and CCI score; we replaced CCI score with CCI items to build model 2. CCI scores and status of CCI items at the end of F/U seems to be more reasonable predictors for overall survival than baseline CCI scores and status of CCI items. Therefore, we employed CCI scores and CCI items at the end of F/U as predictors of overall survival. We found that he enzalutamide group had a lower risk of overall mortality compared with abiraterone in both model 1 (aHR = 0.71, 95% CI: 0.57–0.88, *p* = 0.002) and model 2 (aHR = 0.68, 95% CI: 0.55–0.84, *p* < 0.0001). Other significant predictors of overall survival in the models were age (model 1: aHR = 1.44, 95% CI: 1.13–1.85, *p* = 0.004; model 2: aHR = 1.38, 95% CI: 1.07–1.78, *p* = 0.014) and chemotherapy (model 1: aHR = 1.72, 95% CI: 1.39–2.14, *p* ≤ 0.0001; model 2: aHR = 1.87, 95% CI: 1.50–2.35, *p* < 0.0001). In model 1, the CCI score was a significant predictor of overall survival (aHR = 0.78 for score 2; aHR = 0.93 for score 3; aHR = 0.85 for score 4; aHR = 1.46 for score 5; and aHR = 1.64 for score ≥6, *p* = 0.021). In model 2, we found that no CCI items (i.e., diabetes, congestive heart failure, peripheral vascular disease, chronic pulmonary disease, cerebrovascular disease, moderate or severe renal disease, ulcer disease, mild liver disease, myocardial infarct, AIDS, diabetes with end organ complications, dementia, hemiplegia, connective tissue disease, moderate severe liver disease) were statistically significant predictors of overall mortality (Table 3). In conclusion, age, chemotherapy, CCI score, and abiraterone or enzalutamide use were significant predictors of overall survival. Kaplan-Meier survival curves for different factors.

**TABLE 3:** Predictors of Overall survival, analyzed by Cox Hazard Ration Regression with model 1(CCI scores) and model 2 (CCI items).

Variables		Model 1		Model 2	
		Adjusted HR (95%CI)	*p*	Adjusted HR (95%CI)	*p*
**Group**					
Abiraterone	Ref	1		1	
Enzalutamide		0.71 (0.57–0.88)	**0.002**	0.68 (0.55–0.84)	**<0.0001**
**Age**					
<65	Ref	1		1	
≥65		1.44 (1.13–1.85)	**0.004**	1.38 (1.07–1.78)	**0.014**
**Chemotherapy**					
Naïve	Ref	1		1	
Received		1.72 (1.39–2.14)	**<0.0001**	1.87 (1.50–2.34)	**<0.0001**
**Grade**					
1	Ref	1		1	
2		1.17 (0.55–2.49)	0.670	1.16 (0.54–2.46)	0.553
3		1.36 (0.67–2.76)		1.37 (0.68–2.79)	
Unknow		1.39 (0.64–3.01)		1.47 (0.68–3.20)	
**CCI**					
0	Ref	1		-	-
2		0.78 (0.29–2.10)	**0.021**	-	-
3		0.93 (0.40–2.17)		-	-
4		0.85 (0.36–2.04)		-	-
5		1.46 (0.63–3.34)		-	-
6+		1.64 (0.84–3.20)		-	-
**CCI Items at the end of F/U**	(ref: No)			1	
Diabetes		-	-	0.93 (0.71–1.22)	0.586
CHF		-	-	0.98 (0.73–1.31)	0.893
PVD		-	-	1.34 (0.93–1.93)	0.114
CPD		-	-	1.11 (0.89–1.40)	0.351
Cerebrovascular disease		-	-	1.24 (0.95–1.62)	0.111
Ulcer disease		-	-	0.85 (0.67–1.06)	0.142
Mild liver disease		-	-	1.00 (0.74–1.35)	0.989
MI		-	-	1.49 (1.00–2.23)	0.051
AIDS		-	-	1.18 (0.57–2.46)	0.651
Diabetes with end organ		-	-	1.06 (0.71–1.57)	0.788
Dementia		-	-	0.81 (0.45–1.43)	0.459
Hemiplegia		-	-	1.45 (0.69–3.05)	0.330
CTD		-	-	0.85 (0.67–1.06)	0.249
Moderate severe renal disease		-	-	1.29 (1.00–1.66)	**0.050**
Moderate severe liver disease		-	-	1.32 (0.41–4.26)	0.645

CPD, Chronic pulmonary disease; PVD, Peripheral vascular disease; CTD, Connective tissue disease; MI, Myocardial infarct; CHF, Congestive heart failure; CKD, Chronic Kidney disease; ACS, Acute Coronary Syndrome. Ref: reference group. Bold values mean significantly related.

**FIGURE 2:**
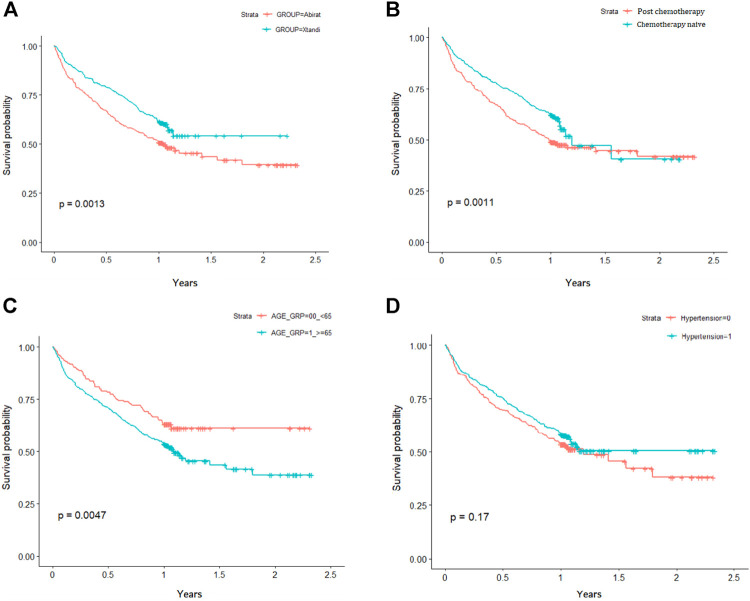
Kaplan-Meier analysis for overall survival, stratified by **(A)** abiraterone/enzalutamide **(B)** chemotherapy **(C)** age; and **(D)** hypertension status.

Kaplan-Meier survival plots showed the risk of overall survival probability between abiraterone and enzalutamide groups. After matching, the enzalutamide group had a lower risk of death than the abiraterone group (log-rank test *p* = 0.0013; Figure 2A). In addition, patients who underwent chemotherapy treatment (log-rank test *p* = 0.0011; Figure 2B) and those who were less than age 65 years (log-rank test *p* = 0.0047; Figure 2C) had significantly survival. However, no significant difference in the effect of hypertension on the cumulative probability of survival was observed between the cohorts (log-rank test *p* = 0.17; Figure 2D).

## Discussion

Our results revealed that age, chemotherapy use, hypertension, duration of follow-up, and duration of medication differed between hormone treatment groups. To compare newly developed comorbidities between abiraterone and enzalutamide, we used a propensity score to match the differing variables. Propensity score matching is a well-known method to reduce bias from treatment assignment and mimic randomization in observational studies, when treatment assignment is not random. Propensity score matching has been used successfully in studies about prostate cancer. Yasukawa et al. (2020) used a propensity score to compare abiraterone plus prednisolone vs. combined androgen blockade in high-risk metastatic, hormone-sensitive prostate cancer. Wu et al. (2020) conducted a nationwide longitudinal cohort study; by adjusting for age and other comorbidities with propensity score matching, this study confirmed an incremental risk of developing hypertension after hormone therapy for prostate cancer. In that study, the cohort that had received androgen deprivation therapy at any time was 1.78 times more likely to develop new-onset hypertension than the control group (Wu et al., 2020). Another observational study that compared stereotactic body radiation therapy and high-dose-rate brachytherapy boost in combination with intensity-modulated radiation therapy for localized prostate cancer also used propensity score matching to minimize bias from the treatment assignment (Chen et al., 2020). In this study, we attempted to compare newly developed comorbidities and survival in patients treated with abiraterone or enzalutamide. The existing confounders of age, chemotherapy, hypertension, duration of follow-up, and duration of medication use were statistically significantly different in these two groups of patients. Furthermore, a causal relationship between these confounding factors and comorbidities has been well established—for example, between age and comorbidities (Piccirillo et al., 2008), age and overall survival (Piccirillo et al., 2008), hypertension and cerebrovascular accident (Meschia et al., 2014), chemotherapy and survival (Chowdhury et al., 2020), hypertension and life expectancy (Turin et al., 2012), and hypertension and cardiovascular disease (Francula-Zaninovic and Nola, 2018).

We also used the duration of medication use to confirm the hypothesis that medication exposure time is correlated to accumulation of a comorbidity; this result has been observed in another study of patients receiving androgen deprivation therapy (O’Farrell et al., 2015). After propensity score matching, we found statistically significant differences in overall survival. The baseline statuses of factors precipitating the development of comorbidities were similar between the abiraterone and enzalutamide groups, even when CCI scores were statistically significantly different between the groups.

Our results after propensity score matching that adjusted the analysis for age, chemotherapy status, and hypertension suggested that overall survival is better with enzalutamide than with abiraterone. Some studies support this finding. Al-Ali et al. (2018) extracted outcomes related to abiraterone and enzalutamide treatment from a medical claims database. Their work showed that the median overall survival of the entire cohort was 21 months, but it was 15 months for abiraterone, 24 months for enzalutamide, and 26 months for the sequence group (Al-Ali et al., 2018). In the post-chemotherapy cohort, enzalutamide provided better overall survival than abiraterone, but not in the pre-chemotherapy cohort. Sathianathen et al. (2020) conducted a meta-analysis of data from metastatic hormone-sensitive prostate cancer and reported that enzalutamide plus androgen deprivation therapy, vs. androgen deprivation therapy alone, had the lowest adjusted HR. Compared with other combination therapies or with androgen deprivation therapy alone, enzalutamide plus androgen deprivation therapy was the preferred treatment to prolong overall survival (Sathianathen et al., 2020).

However, some studies had different findings. A more recent meta-analysis of 15 cohort studies involving 3546 participants showed that the PSA response rate—but not overall survival—was significantly greater in the enzalutamide group than in the abiraterone group (Wang et al., 2020). In an earlier study, Zhang et al. (2017) announced that indirect comparisons between abiraterone and enzalutamide in mCRPC showed no statistically significant difference in overall survival in pre-chemotherapy and post-chemotherapy settings. However, enzalutamide outperformed abiraterone in secondary endpoints, including time to PSA progression, radiographic progression-free survival, and PSA response rate (Zhang et al., 2017). Chopra et al. (2017) reported that enzalutamide outperformed abiraterone plus prednisone in terms of radiographic progression-free survival, PSA progression, and PSA response rate, but not in terms of overall survival, in the pre- and post-docetaxel settings. A study conducted by Tan et al. (2014) shared the same results and found no statistically significant difference in overall survival, despite the potential advantage of enzalutamide for secondary endpoints. Generally speaking, most authors agree that enzalutamide yields better oncological outcomes. However, the studies described in this section did not consider significant comorbidities. In our study, we controlled for the confounding variables of age and comorbidity, which have accepted influences on overall survival, to compare the overall survival between hormone treatment groups. The univariable analysis found that enzalutamide was superior to abiraterone plus prednisolone. The Kaplan-Meier analysis also showed a statistically significant better survival in the enzalutamide group. Furthermore, enzalutamide had a lower adjusted HR than abiraterone in Cox regression analysis. These findings are valuable details that can guide clinical decision making and medication selection.

We found no statistically significant difference in newly developed comorbidities, including cerebrovascular disease, peripheral vascular disease, peptic ulcer disease, congestive heart failure, chronic renal disease, myocardial infarction, diabetes mellitus, dementia, and acute coronary syndrome. Few studies comprehensively compare comorbidities between abiraterone and enzalutamide. However, some comorbidities, such as congestive heart failure (Bretagne et al., 2020) and cardiovascular disease (Lee et al., 2020), have been connected with abiraterone and enzalutamide treatment. Bretagne et al. (2020) conducted an observational, retrospective pharmacovigilance study and found that abiraterone was associated with arrhythmia and heart failure (Bretagne et al., 2020). Conversely, a systematic review with pairwise and network meta-analyses demonstrated that abiraterone and enzalutamide cause different cardiovascular comorbidities. The researchers found that enzalutamide was associated with increased risks of any grade of hypertension, whereas abiraterone increased the probability of cardiac disorders (Lee et al., 2020). Some indirect evidence suggests that abiraterone and enzalutamide may be associated with dementia or diabetes with end organ complications.

A review of treatments for prostate cancer and their impacts on the central nervous system and cognitive function showed that enzalutamide was associated with more amnesia, cognitive disorders, memory impairment, confused states, and fatigue than abiraterone (Ryan et al., 2020). No direct evidence exists to confirm an association of abiraterone or enzalutamide with the development of dementia. Tucci et al. (2019) reported that abiraterone plus prednisone induced severe hypoglycemia in patients with type 2 diabetes who were receiving glucose-lowering agents (e.g., sulfonylureas). However, results did not confirm an association between abiraterone plus prednisone or enzalutamide and the development of diabetes mellitus (Tucci et al., 2019). No clinical studies have described possible nephrotoxic effects from abiraterone acetate, and the safety of abiraterone and enzalutamide has been established in patients with renal impairment (Meschia et al., 2014). To our knowledge, no evidence connects these new-generation secondary hormone therapies with cerebrovascular disease, peripheral vascular disease, or peptic ulcer disease. As we know, age, hypertension, hyperlipidemia, and diabetes mellitus are strong precipitating factors for cardiovascular disease and cerebrovascular disease (Meschia et al., 2014; Yan et al., 2017). We adjusted the analysis in our study by using propensity scores for factors that were statistically different between groups. Thus, we adjusted the baseline age and hypertension with propensity score matching so that we could eliminate the bias of unequal status between abiraterone and enzalutamide. Our results provided evidence of the equality of comorbidity development between abiraterone and enzalutamide; these results will help guide clinical decision making and determine medication selection.

In the Cox regression analysis for the endpoints of overall survival and disease-specific survival, we found that age, chemotherapy status, abiraterone or enzalutamide use, and CCI score—but not CCI items—were significant predictors of overall survival. Our data suggested that previous chemotherapy use is a negative predictor of overall survival. The negative association most likely resulted from that the failure of post-chemotherapy was an essential inclusion criteria required by the pre-examination operation for payments between September 2016 and August 2017. Since September 2017, health insurance has permitted claims for chemotherapy-naive mCRPC. Thus, patients with a post-chemotherapy status in our dataset tended to be in a later disease stage than chemotherapy-naive patients. Therefore, chemotherapy status was a confounding variable that required elimination to ensure equality of baseline characteristics between the groups.

We demonstrated that the CCI score, rather than any single item of the CCI, predicted overall survival for patients with mCRPC who received abiraterone or enzalutamide treatment. Some researchers reported opposite findings. One study using androgen deprivation therapy showed that the CCI did not predict overall survival independent of known prognostic factors in mCRPC (Goyal et al., 2014). Zist et al. (2015) conducted a study to clarify the impacts of comorbidity on outcomes in men with advanced prostate cancer treated with docetaxel and found a higher CCI was not associated with worse overall survival. However, some studies reported findings consistent with ours. Karel et al. (Vanh and Vreugdenhil, 2019) discovered that an age-adjusted CCI strongly influenced overall survival, irrespective of performance status, in patients with advanced prostatic cancer treated with enzalutamide. Ording et al. conducted a Danish cohort study of 45,326 patients with prostate cancer who were diagnosed between 1995 and 2011. The study found that an interaction between comorbidity and prostate cancer, which explained up to 20% of all deaths, was present for patients with metastatic disease and those not treated with prostatectomy (Ording et al., 2016). Our results suggested that the CCI score, rather than single items in the CCI, is a strong predictor for overall survival. This phenomenon could be explained by death more often resulting from an accumulation of comorbidities rather than a single comorbidity.

Our study has two noteworthy strengths. First, we used the nationwide NHIRD as the source of study material. This database contains information on 99% of the population in Taiwan, and the pre-review counter for abiraterone and enzalutamide is executed with strict criteria by experienced urologists, so selection bias was minimized. Second, we comprehensively compared the incidence of newly developed comorbidities in patients with mCRPC who were treated with abiraterone or enzalutamide after propensity score matching for precipitating factors. We ensured the equality of baseline characteristics between groups. However, this study also had limitations. First, our database could not provide enough data for secondary endpoints, such as PSA response rate, progression-free survival, or radiographic progression-free survival. Second, we could not assess the outcomes, adverse effects, or comorbidities associated with the sequencing use of abiraterone and enzalutamide, though this approach has been heatedly discussed recently. A well-designed, head-to-head, randomized, controlled trial is warranted to clarify the association among survival, newly developed comorbidities, and CCI in patients with mCRPC who are treated with abiraterone or enzalutamide.

## Conclusion

We concluded after propensity score matching that the development of new comorbidities is not affected by the medication choice from the new generation of secondary hormone therapies (i.e., abiraterone or enzalutamide). We also found the overall survival was better in the enzalutamide than in the abiraterone group after analysis was adjusted for age, CCI, and chemotherapy status. Furthermore, CCI score overall, rather than its items, was a statistically significant predictor of overall survival. Our findings provide evidence about the association between CCI and clinical outcomes and could help guide clinical decision making and medication selection.

## Data Availability

The data analyzed in this study is subject to the following licenses/restrictions: NHIRD provides de-identification demographic and administrative information, all NHIRD data must be reviewed and processed by the research project before analysis. Requests to access these datasets should be directed to https://dep.mohw.gov.tw/dos/np-1714-113.html.
